# Junction-Associated Laminin-R/RPSA Immunoreactivity in Electrical Marks: A Morphological and Immunohistochemical Study

**DOI:** 10.3390/ijms27198872

**Published:** 2026-10-05

**Authors:** Anna Claudia Caruso, Damiano Marchesini, Anna Maria Manta, Donato Morena, Matteo Scopetti, Martina Padovano, Roberta Bibbò, Luigi Cipolloni, Vittorio Fineschi

**Affiliations:** 1 Department of Anatomical, Histological, Forensic and Orthopedic Sciences, Sapienza University of Rome, 00161 Rome, Italy; damiano.marchesini@uniroma1.it (D.M.); annamaria.manta@uniroma1.it (A.M.M.); donato.morena@uniroma1.it (D.M.); vittorio.fineschi@uniroma1.it (V.F.); 2 Department of Medical Surgical Sciences and Translational Medicine, Sapienza University of Rome, 00189 Rome, Italy; matteo.scopetti@uniroma1.it; 3 Department of Public Security, Directorate for the Forensic Science Police and the Cyber Security, Forensic Science Police Service, 00173 Rome, Italy; martina.padovano@uniroma1.it; 4 Department of Clinical and Experimental Medicine, Section of Forensic Medicine, University of Foggia, 71122 Foggia, Italy; roberta.bibbo@unifg.it (R.B.); luigi.cipolloni@unifg.it (L.C.)

**Keywords:** electrocution, electrical mark, dermo-epidermal junction, Laminin-R, RPSA, immunohistochemistry, forensic pathology

## Abstract

Cutaneous electrical marks may provide the principal localized morphological evidence of contact with an electrical conductor, but their histopathological interpretation remains challenging. We investigated the topographical relationship between dermo-epidermal detachment and junction-associated Laminin-Receptor/Ribosomal Protein SA (Laminin-R/RPSA) immunoreactivity in 25 full-thickness skin specimens from documented fatal electrocution cases. Detachment on hematoxylin and eosin (H&E) staining was initially classified as present, absent, or equivocal, with discordant or equivocal assessments subsequently adjudicated by a supervising observer. After adjudication, detachment was present in 22 specimens (88%) and absent in three (12%). All 22 specimens with detachment lacked detectable junction-associated Laminin-R/RPSA immunoreactivity at the corresponding interface, whereas all three specimens without detachment retained the signal. All 25 preserved perilesional regions showed retained immunoreactivity. The paired absolute difference in the proportion showing loss of immunoreactivity between lesion and perilesional sites was 88.0 percentage points (95% CI, 55.5–97.5; exact McNemar *p* < 0.0001). These findings demonstrate a consistent spatial correspondence between dermo-epidermal separation and localized loss of detectable Laminin-R/RPSA immunoreactivity. Because the antibody targets RPSA rather than laminin-332, the results do not directly establish laminin-332 damage. Laminin-R/RPSA immunohistochemistry may complement conventional H&E examination, but controlled comparative studies are required to determine specificity and reproducibility.

## 1. Introduction

Electrical injuries remain an important cause of morbidity and mortality, particularly after domestic and occupational exposure. The extent and distribution of tissue injury depend on multiple interacting variables, including current type and intensity, voltage, tissue resistance, current pathway, contact surface, and duration of exposure [1]. At the contact site, the high resistance of the skin influences both the amount and localization of heat generated within the tissues [2].

In forensic practice, particular attention is directed toward the cutaneous electrical mark, which may constitute the only external lesion providing direct morphological support for contact between the skin and an electrical conductor. Its absence, however, does not exclude a fatal electrical event. Internal autopsy findings are generally nonspecific because death commonly results from cardiac arrhythmia or respiratory arrest. Diagnosis therefore requires integration of scene and circumstantial information, technical evaluation of the electrical source, autopsy findings, and histopathological examination [1,3,4,5].

On routine histology, electrical marks may show epidermal coagulative injury, intraepidermal clefting, dermo-epidermal detachment, nuclear hyperchromasia, pyknosis, and elongation or streaming of epidermal nuclei [2,4]. These findings are supportive but not individually pathognomonic, as partially overlapping changes may occur after thermal exposure, tissue desiccation, or histological processing [3,4]. Ancillary approaches, including metallization studies, scanning electron microscopy, energy-dispersive X-ray analysis, and microbeam X-ray fluorescence, may strengthen the assessment in selected cases but do not replace integrated forensic evaluation [6,7,8]. Immunohistochemistry offers an additional means of examining spatially localized tissue-antigen patterns in relation to lesions identified on H&E staining. Molecular and immunohistochemical studies of cutaneous electrical injury remain limited [9].

The epidermis and dermis meet at the dermo-epidermal junction, a specialized adhesion interface formed by basal keratinocytes, hemidesmosomal complexes, anchoring filaments, the basement-membrane zone, and anchoring fibrils. Ultrastructurally, this interface includes the lamina lucida, lamina densa, and deeper lamina fibroreticularis [10]. This highly organized architecture is directly relevant to the interpretation of dermo-epidermal detachment.

Laminins are extracellular-matrix glycoproteins associated with basement membranes [11]. At the dermo-epidermal junction, they participate in a complex adhesion system involving integrin α6β4, type VII collagen-containing anchoring structures, and other basement-membrane components, thereby supporting stable epidermal attachment and tissue organization [12]. More broadly, extracellular-matrix components contribute to cell–matrix adhesion, cellular signaling, migration, and tissue repair [12,13].

The antibody employed in the present study is directed against Laminin Receptor/Ribosomal Protein SA (Laminin-R/RPSA), also known as the 37/67-kDa non-integrin laminin receptor. RPSA is a multifunctional protein found in ribosomal and other intracellular compartments and, in some contexts, at the cell surface, where it can interact with laminin [14].

The selection of RPSA was supported by both technical feasibility and biological relevance. The availability of an antibody suitable for immunohistochemical application to routinely processed archival tissue enabled its assessment in a retrospective series, while its laminin-binding properties and context-dependent cell-surface localization made its junction-associated distribution potentially informative in relation to dermo-epidermal cleavage [12,14]. Although exploratory, this approach provides complementary information on the local organization of the laminin–receptor interface and its spatial relationship with morphological separation.

The present investigation therefore focused on the topographical relationship between dermo-epidermal detachment and junction-associated Laminin-R/RPSA immunoreactivity in documented electrical marks. The primary aim was to determine whether detachment identified on H&E staining was associated with loss of detectable Laminin-R/RPSA immunoreactivity at the anatomically corresponding junctional site. A secondary aim was to determine whether the lesion-associated staining pattern differed from morphologically preserved perilesional skin within the same specimens.

## 2. Results

### 2.1. Histological and Immunohistochemical Findings

All 25 specimens were evaluable for the combined histological and immunohistochemical assessment. After supervisor adjudication, dermo-epidermal detachment was classified as present in 22 specimens (88.0%) and absent in three specimens (12.0%). Junction-associated Laminin-R/RPSA immunoreactivity was absent in 22 specimens (88.0%) and detectable in three specimens (12.0%). All 22 specimens with dermo-epidermal detachment showed focal interruption or absence of detectable Laminin-R/RPSA immunoreactivity at the anatomically corresponding junctional region, whereas all three specimens without detachment retained junction-associated immunoreactivity.

Before adjudication, the two primary observers agreed on H&E classification in 24 of 25 specimens (96.0%; exact 95% CI, 79.6–99.9%), with an unweighted Cohen’s κ of 0.841. Agreement for junction-associated Laminin-R/RPSA immunoreactivity was likewise observed in 24 of 25 specimens (96.0%; exact 95% CI, 79.6–99.9%), with an unweighted Cohen’s κ of 0.834.

**Figure 1:**
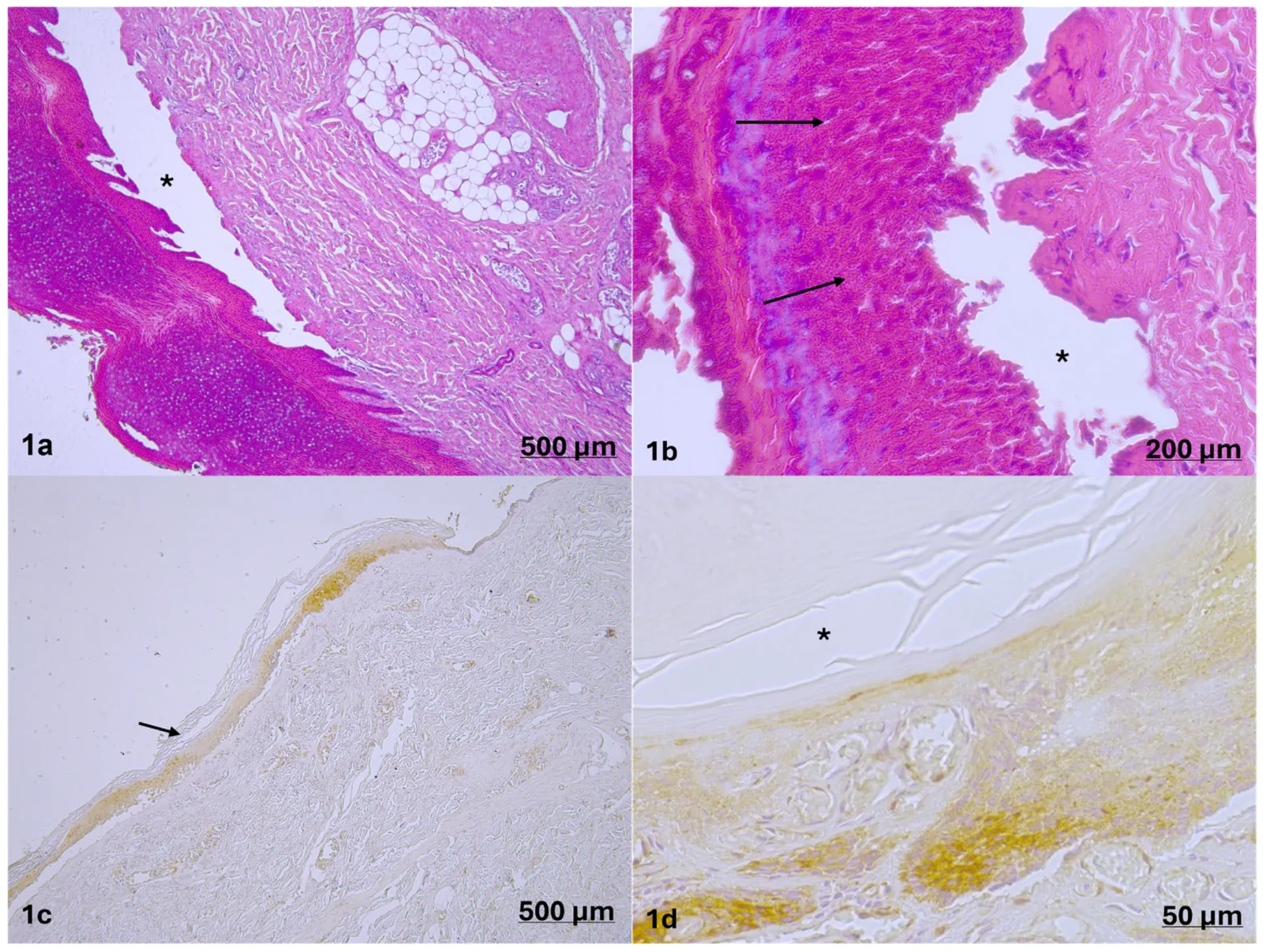
Representative histological and immunohistochemical findings in electrical marks. (**1a**) Low-power hematoxylin and eosin (H&E) view showing extensive dermo-epidermal separation (asterisk) (scale bar: 500 µm). (**1b**) Higher-magnification H&E view showing dermo-epidermal separation (asterisk) and elongation and streaming of epidermal nuclei (arrows) (scale bar: 200 µm). (**1c**) Low-power Laminin-R/RPSA immunostaining showing absence of detectable junction-associated immunoreactivity along the detached epidermal interface (arrow) (scale bar: 500 µm). (**1d**) Higher-magnification Laminin-R/RPSA immunostaining showing a dermo-epidermal detachment (asterisk), with absence of detectable immunoreactivity along the detached epidermal edge and retained linear immunoreactivity along the subjacent tissue interface (scale bar: 50 µm).

All 25 morphologically preserved perilesional regions retained junction-associated Laminin-R/RPSA immunoreactivity. The transition between a preserved epidermal region and a detached interface was accompanied by a corresponding transition from retained to interrupted immunoreactivity, supporting a localized pattern rather than nonspecific staining failure throughout the section. Representative findings are shown in Figure 1. Figure 1c,d show focal loss of expression of Laminin-R/RPSA, with dermo-epidermal separation in the immediate vicinity, while linear immunoreactivity is still present in the adjacent area.

### 2.2. Paired Lesion–Perilesional Analysis

The final adjudicated lesion-site findings are summarized in Table 1. All 22 specimens with dermo-epidermal detachment showed absence of detectable junction-associated Laminin-R/RPSA immunoreactivity, whereas all three specimens without detachment retained the signal. The association between final H&E classification and Laminin-R/RPSA immunoreactivity was statistically significant (two-sided Fisher’s exact *p* = 0.0004348).

In the paired analysis, junction-associated immunoreactivity was absent at the lesion site in 22 of 25 specimens (88.0%) and in none of the 25 matched morphologically preserved perilesional regions. There were 22 discordant pairs with lesion-site loss/perilesional retention, no discordant pairs in the opposite direction, and three concordant positive pairs. The paired absolute difference in the proportion showing loss of immunoreactivity was 0.88 (95% CI, 0.555–0.975), corresponding to 88.0 percentage points (95% CI, 55.5–97.5). The exact McNemar test yielded *p* < 0.0001.

Given the small number of specimens classified as having no dermo-epidermal detachment after adjudication, Fisher’s exact test should be interpreted together with the absolute counts and the observed topographical correspondence. The analysis demonstrates an association between final H&E classification and Laminin-R/RPSA staining status within this series, but does not establish specificity for electrical injury or for dermo-epidermal detachment across other lesion types.

## 3. Discussion

### 3.1. Principal Finding and Biological Interpretation

The principal finding was the close topographical correspondence between dermo-epidermal detachment and loss of detectable junction-associated Laminin-R/RPSA immunoreactivity in cutaneous electrical marks. After adjudication, all 22 specimens classified as showing dermo-epidermal detachment showed interruption or absence of the immunohistochemical signal at the anatomically corresponding interface, whereas all three specimens classified as having no detachment retained junction-associated immunoreactivity.

Dermo-epidermal separation is a recognized microscopic feature of electrical injury, together with coagulative changes and elongation or streaming of epidermal nuclei [2,4]. Its occurrence may vary according to voltage, duration of contact, skin resistance, contact surface, anatomical site, and the interval between injury and tissue sampling [1,2]. Fixation, sectioning, tissue orientation, and desiccation may also influence its recognition [3]. The high proportion of specimens showing detachment confirms that it was a prominent morphological feature in the examined series, but does not establish diagnostic specificity.

**Figure 2:**
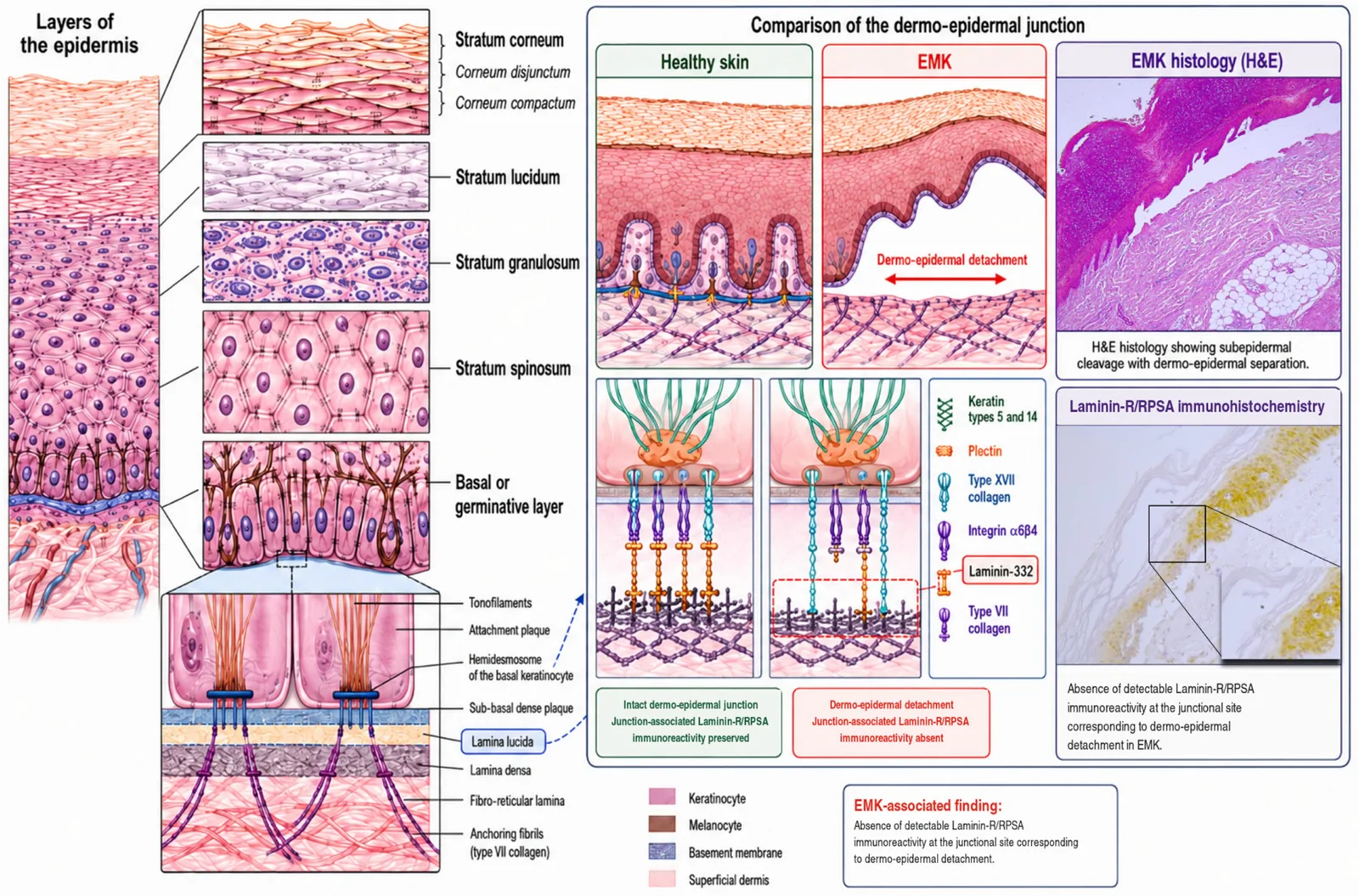
Schematic and histopathological overview of the dermo-epidermal junction in electrical marks. The left panel illustrates the epidermal layers and basement-membrane organization. The central schematic compares intact skin with an electrical mark (EMK), highlighting dermo-epidermal detachment and the principal components of the anchoring complex. Representative hematoxylin and eosin (H&E) staining shows subepidermal cleavage with dermo-epidermal separation (scale bar: 500 µm). Laminin-R/RPSA immunohistochemistry shows absence of detectable junction-associated immunoreactivity at the site corresponding to the detachment (scale bar: 500 µm). The present study investigated Laminin-R/RPSA immunoreactivity, demonstrating a topographical correspondence between loss of detectable staining and dermo-epidermal detachment. The other components of the anchoring complex are illustrated solely as anatomical background and were not directly investigated in the present study.

The biological plausibility of the observed pattern derives from the highly organized adhesive architecture of the dermo-epidermal junction. Stable attachment depends on coordinated interactions among basal keratinocytes, hemidesmosomal proteins, anchoring filaments, basement-membrane components, and anchoring fibrils [10]. Laminin occupies a central position within this network, contributing to the organization, stability, and adhesive properties of the dermo-epidermal junction [11,12]. The laminin-binding properties of RPSA provide a biological rationale for examining its junction-associated distribution [14]. A schematic overview of the dermo-epidermal junction and the relationship between dermo-epidermal detachment and Laminin-R/RPSA immunoreactivity is shown in Figure 2.

Dermo-epidermal separation was identified morphologically by H&E examination. At the corresponding sites, RPSA immunohistochemistry enabled assessment of the distribution and continuity of a laminin-binding receptor-associated signal. Its focal interruption therefore provides a complementary molecular correlate of junctional cleavage and contributes to the characterization of local alterations in the laminin-associated cell–matrix interface.

In this series, dermo-epidermal detachment showed a consistent spatial correspondence with altered RPSA immunoreactivity at the corresponding interface. The retained linear staining along the subjacent tissue interface illustrated in Figure 1d suggests that immunoreactive structures may persist following separation, with a change in their relationship to the detached epidermal edge. This distribution is compatible with retention or displacement of RPSA-bearing structures and does not necessarily imply antigen degradation. The shared processing and staining conditions of lesional and perilesional regions, together with retained perilesional immunoreactivity, support the localized nature of the observed pattern. Potential contributors include changes in antigenicity, epitope masking, physical displacement of immunoreactive structures, and tissue loss during sectioning or processing. These mechanisms are not mutually exclusive and were not directly tested. The findings therefore characterize the relationship between junctional morphology and RPSA signal distribution, while the respective contributions of molecular and physical changes remain to be determined.

### 3.2. Internal Controls and Statistical Interpretation

The retention of junction-associated immunoreactivity in all morphologically preserved perilesional regions supports the technical adequacy of staining and argues against generalized loss of antigen detectability throughout the sections. Interobserver analyses also showed high reproducibility for both morphological and immunohistochemical scoring before adjudication. The final H&E classification was established after supervisor review of discordant or equivocal assessments.

Fisher’s exact test addresses the association between final H&E classification and lesion-site Laminin-R/RPSA immunoreactivity within this series, whereas the exact McNemar analysis addresses the distinct paired question of whether junction-associated immunoreactivity differs between lesion and morphologically preserved perilesional regions within the same specimen. The paired absolute difference further quantifies the magnitude of this within-specimen contrast. Neither analysis establishes diagnostic accuracy, specificity for electrical injury, or specificity for dermo-epidermal detachment across other lesion types. The internal comparator does not substitute for independently sampled unaffected skin or for thermal, mechanical, post-mortem, and processing-related control lesions.

### 3.3. Limitations

This retrospective exploratory study has several limitations. First, the sample was small, and the final H&E categories remained markedly unbalanced, with only three specimens classified as having no dermo-epidermal detachment after adjudication. The retrospective archival design also limited the uniform availability of demographic and exposure-related details, although the available documentation was sufficient to confirm the inclusion criteria in all cases.

Second, no external comparator groups comprising unaffected skin, thermal burns, abrasions, experimentally produced post-mortem electrical lesions, processing artefacts, or other non-electrical lesions with dermo-epidermal separation were included. The present data therefore cannot establish whether the RPSA pattern is specific to electrical injury or accompanies junctional cleavage more generally.

Third, consistent with the exploratory nature of the study, junction-associated immunoreactivity was assessed qualitatively and recorded as present or absent, without quantitative measurement. Future studies could incorporate quantitative digital image analysis to further characterize the intensity and spatial extent of junction-associated staining. Fourth, fixation, antigen retrieval, tissue orientation, sectioning, and physical tissue loss at sites of detachment may have influenced antigen detectability or representation of the interface, although retained staining in paired perilesional regions argues against generalized technical failure.

Specimen selection prioritized interpretable tissue architecture for assessment of the dermo-epidermal interface. Exclusion of advanced putrefaction and prolonged survival associated with local lesion treatment reduced potential confounding by post-mortem alteration and treatment-related changes. Where tissue destruction removes or obscures the target interface, absent immunoreactivity cannot be reliably separated from loss of the anatomical substrate. The present findings therefore concern the selected archival specimens; performance in markedly degraded, charred, or otherwise poorly preserved tissue requires dedicated evaluation.

Finally, within the exploratory scope of the study, marker selection was partly guided by the availability of an antibody suitable for immunohistochemical application to routinely processed archival tissue. Accordingly, the analysis focused on Laminin-R/RPSA, which is biologically distinct from laminin-332. The results therefore do not directly document damage to laminin-332 or other structural junctional proteins. The term “junction-associated Laminin-R/RPSA immunoreactivity” should thus be understood as a topographical description of the observed staining pattern, rather than as evidence that RPSA is itself a structural component equivalent to laminin-332.

### 3.4. Future Directions

Future studies should validate the observation in independent series incorporating unaffected, thermal, mechanical, experimentally produced post-mortem, and processing-related comparator groups. Non-electrical lesions characterized by dermo-epidermal separation will be particularly informative for distinguishing an electrical-injury-associated pattern from a general correlate of junctional cleavage.

Parallel staining for laminin-332 chains, integrin α6β4, type IV collagen, and type VII collagen could determine whether altered RPSA detectability accompanies a broader disruption of the basal keratinocyte-matrix interface. Digital image analysis could quantify signal length, continuity, and intensity. Examination of serial sections, combined with complementary immunolocalization and ultrastructural studies, could further define the position of immunoreactive structures relative to the cleavage plane and help assess the contribution of tissue displacement or loss to the observed staining pattern.

Further validation should assess specimens with different degrees of decomposition and thermal damage, as well as documented survival intervals and local treatments, evaluating both junctional interpretability and preservation of RPSA immunoreactivity.

## 4. Materials and Methods

### 4.1. Study Design and Sample Selection

Twenty-five full-thickness skin specimens, each obtained from a different deceased individual with documented electrocution, were retrieved from the histopathology archives of the Institute of Legal Medicine, Department of Anatomical, Histological, Forensic and Orthopedic Sciences, Sapienza University of Rome, and the Department of Clinical and Experimental Medicine, University of Foggia. Specimens were included only when the diagnosis of electrocution was supported by concordant circumstantial, autopsy, and histopathological findings. Each specimen contained the central portion of a macroscopically documented electrical mark. Cases showing advanced putrefactive changes, prolonged post-injury survival associated with local treatment of the lesion, or mixed cutaneous lesions that could not be reliably interpreted were excluded. Morphologically preserved perilesional skin, located approximately 1 cm from the macroscopically identified electrical mark at the margins of the same full-thickness specimen, served as an internal paired comparator. These regions showed preserved epidermal architecture and an intact dermo-epidermal interface on H&E examination, without unequivocal detachment in the area assessed. This comparison allowed evaluation of junction-associated Laminin-R/RPSA immunoreactivity under shared fixation, processing, and staining conditions. Morphological preservation was not taken to imply absence of electrical or thermal exposure, and these regions were used as within-specimen comparators rather than independently sampled normal skin.

The series was retrospectively assembled from archival histological material. Paraffin blocks and stained slides were available for all 25 cases. Although the available records were sufficient to verify the inclusion criteria, complete and standardized case-level data were not consistently retrievable for all specimens. Age, sex, exact anatomical site, post-mortem and survival intervals, voltage category, current type, contact duration, and detailed exposure circumstances could therefore not be reported uniformly or included in stratified analyses.

### 4.2. Histological Examination

Skin fragments were fixed in 10% neutral-buffered formalin for more than 24 h, routinely processed, and embedded in paraffin. Section 3 and Section 4 µm thick were stained with H&E. For the purposes of the present analysis, review of the routine sections was restricted to dermo-epidermal detachment.

Dermo-epidermal detachment was defined as separation between the epidermal basal layer and the underlying superficial dermis/basement-membrane region. It was classified as present when a clear and morphologically interpretable separation was identified, absent when the junction remained preserved, and equivocal when focal separation could not be distinguished with confidence from tissue-preservation or processing-related change.

### 4.3. Immunohistochemistry

Immunohistochemical analysis was performed on 4-µm paraffin sections mounted on 3-aminopropyltriethoxysilane-coated slides. A mouse monoclonal anti-Laminin-R/RPSA antibody, clone G-7 (Santa Cruz Biotechnology, cat. no. sc-74531), raised against amino acids 110–250 of human Laminin-R/RPSA, was applied at a dilution of 1:50.

Sections were deparaffinized in xylene and rehydrated through graded alcohols. Heat-induced epitope retrieval was performed in 10 mM citrate buffer, pH 6.0, at 95 °C for 5 min, followed by replenishment with fresh buffer and a second 5-min heating step. Slides were allowed to cool in buffer for approximately 20 min and were then washed three times in deionized water. After blocking nonspecific reactivity, sections were incubated with the primary antibody and processed using an HRP-polymer detection system with 3,3′-diaminobenzidine as chromogen. Sections were counterstained with Mayer’s hematoxylin, dehydrated, and coverslipped.

All sections were examined using a Leica DM4000 B light microscope connected to a Leica DC480 digital camera system under transmitted bright-field illumination. External positive controls consisted of intact epidermis with expected detectable Laminin-R/RPSA immunoreactivity, providing a reference for antigen detection in epidermal tissue. Negative controls were prepared by omitting the primary antibody to assess staining arising independently of primary-antibody application. Morphologically preserved epidermal regions within each electrical-mark section served as internal positive controls, and their preserved dermo-epidermal interfaces provided a within-section reference for the assessment of junction-associated immunoreactivity.

### 4.4. Topographical Assessment and Statistical Analysis

All H&E-stained and corresponding Laminin-R/RPSA-immunostained sections were independently evaluated by two observers (A.M.M. and A.C.C.) experienced in forensic histopathology. Cases with discordant assessments or equivocal H&E findings were subsequently reviewed by a supervising observer (V.F.), whose assessment was used for the final adjudicated classification. After classification of detachment on H&E, the anatomically corresponding junctional region was examined on the immunohistochemical section. Detectable Laminin-R/RPSA immunoreactivity was recorded as present (+) or absent (-). Assessment was restricted to immunoreactivity at the dermo-epidermal interface identified by anatomical correspondence with the H&E section. Lesion-site staining was interpreted relative to the signal at morphologically preserved junctional regions within the same immunostained section. Generalized cytoplasmic reactivity remote from the interface was excluded from the junction-associated assessment, irrespective of its intensity. This exclusion was anatomical rather than an assumption that cytoplasmic staining was nonspecific, since RPSA also has intracellular localization. Accordingly, “junction-associated immunoreactivity” denotes the topographical distribution of the signal assessed. The assessment was not formally blinded to the nature of the study cases.

Absolute frequencies and proportions were calculated for dermo-epidermal detachment and junction-associated Laminin-R/RPSA immunoreactivity. Interobserver reproducibility before adjudication was assessed separately for H&E classification and junction-associated Laminin-R/RPSA immunoreactivity using observed percentage agreement and unweighted Cohen’s kappa. Exact 95% confidence intervals for observed agreement were calculated using the Clopper–Pearson binomial method. After supervisor adjudication, the association between final H&E classification and lesion-site Laminin-R/RPSA immunoreactivity was assessed using a two-sided Fisher’s exact test because of the small sample size and zero cell counts. The paired comparison between lesion-site and morphologically preserved perilesional immunoreactivity within the same specimen (*n* = 25) was assessed using a two-sided exact McNemar test. The paired absolute difference in proportions and its exact 95% confidence interval were additionally calculated. Statistical significance was set at two-sided *p* < 0.05. All analyses were performed in R, version 4.6.1 (R Foundation for Statistical Computing, Vienna, Austria). Observed-agreement confidence intervals and Fisher’s exact test were obtained using the stats package (binom.test and fisher.test), Cohen’s kappa using the irr package (kappa2), and the exact McNemar test with the corresponding confidence interval for the paired difference in proportions using the exact2x2 package (mcnemarExactDP). Given the exploratory design and small sample, these analyses were not interpreted as estimates of diagnostic sensitivity, specificity, or etiological specificity.

## 5. Conclusions

Analysis of 25 documented electrical marks showed dermo-epidermal detachment in 22 specimens (88%) after adjudication. In all 22 specimens with detachment, Laminin-R/RPSA immunoreactivity was undetectable or interrupted along the corresponding junctional region, whereas the signal remained preserved in all three specimens without detachment and in all matched morphologically preserved perilesional regions. The paired lesion–perilesional analysis supported the localized nature of this pattern.

Combining routine morphology with immunohistochemistry therefore demonstrated a consistent spatial correspondence between structural cleavage and alteration of the junction-associated RPSA pattern. Laminin-R/RPSA immunohistochemistry may serve as an adjunct to conventional H&E examination for characterizing electrical marks within an integrated forensic assessment [15]. However, external comparative validation and direct staining of junctional proteins are needed before diagnostic specificity or routine applicability can be established. Future studies could also incorporate quantitative digital image analysis to further characterize junction-associated staining.

## Figures and Tables

**Table 1:** Correspondence between dermo-epidermal detachment and junction-associated Laminin-R/RPSA immunoreactivity (*n* = 25).

H&E Classification	RPSA Absent, n	RPSA Present, n	Total, n
Detachment present	22	0	22
Detachment absent	0	3	3
Total	22	3	25

Final H&E classification reflects supervisor adjudication of discordant or equivocal primary assessments. The association between H&E classification and RPSA immunoreactivity was assessed using Fisher’s exact test.

## Data Availability

The dataset generated and analyzed during the current study is available from the corresponding author upon reasonable request.

## Abbreviations

The following abbreviations are used in this manuscript:

H&E	Hematoxylin and Eosin
EMK	Electric Mark
Laminin-R	Laminin Receptor
RPSA	Ribosomal Protein SA
